# Long-Term Impact of Physical Activity Levels After High-Speed Resistance Training on Cardiac Autonomic Control in Independent Older Adults

**DOI:** 10.1177/30495334251369446

**Published:** 2025-08-31

**Authors:** Alexandre Duarte Martins, Orlando Fernandes, João Paulo Brito, Rafael Oliveira, Bruno Gonçalves, Nuno Batalha

**Affiliations:** 1 Universidade de Évora, Portugal; 2 Santarém Polytechnic University, Portugal

**Keywords:** aging, physical activity, exercise, heart rate variability, cardiovascular system

## Abstract

This study investigated the influence of physical activity (PA) levels on the long-term retention of the effects on cardiac autonomic control, assessed by heart rate variability (HRV), following a 16-week high-speed resistance training (HSRT) program over a 12-month follow-up period. At 12-month follow-up period, 36 participants who completed the measurements were categorized into light activity group (LAG) and moderate-to-vigorous activity group (MVAG) based on their PA levels. HRV data were recorded over a 6-min period. Significant within-group differences were observed over time. In MVAG, systolic blood pressure was significantly lower at the 6-month follow-up compared to pre-intervention (*d*_unb_ = −0.87), while in LAG, diastolic blood pressure was lower at the 12-month follow-up compared to post-intervention (*d*_unb_ = −0.66). Additionally, MVAG demonstrated significantly higher mean RR values at the 12-month compared to the 6-month follow-up (*d*_unb_ = 0.49). Moreover, minimum and mean heart rate values were significantly higher at the 6-month compared to the 12-month follow-up (*d*_unb_ = −0.39 and *d*_unb_ = −0.42, respectively) in MVAG. Lastly, Sample Entropy was significantly higher in LAG compared to MVAG at 12-month follow-up (*d*_unb_ = −0.89). In conclusion, participants who maintained moderate-to-vigorous PA during the 12-month follow-up demonstrated better retention of parasympathetic-related indices effects, as well as overall autonomic nervous system markers.

## Introduction

Cardiovascular diseases (CVD), including heart failure and stroke, remain the leading cause of mortality worldwide (Roth et al., 2020). According to Kannel (2000), major risk factors for CVD include increased blood pressure, glucose intolerance, dyslipidaemia, abdominal obesity, and left ventricular hypertrophy, factors that persist as key contributors to CVD risk (Paluch et al., 2024). Additionally, aging has been linked to a heightened risk of CVD (Díez-Villanueva et al., 2022), largely due to macrovascular and microvascular impairments (Nowak et al., 2018).

Heart rate variability (HRV), a non-invasive method to assess the autonomic nervous system (ANS) function (Shaffer & Ginsberg, 2017; Task Force of the European Society of Cardiology and the North American Society of Pacing and Electrophysiology, 1996), has been linked to CVD risk factors (Fang et al., 2020). It also exhibits dynamic balance between the sympathetic and parasympathetic branches of the ANS, as assessed by variations in beat-to-beat intervals through both linear and non-linear analytical methods (Shaffer & Ginsberg, 2017). Impairments in ANS regulation have been associated with adverse health outcomes in older adults, including functional decline (Ogliari et al., 2015), and sarcopenia (Freitas et al., 2018).

The *American Heart Association* (AHA) has also emphasized physical inactivity as a well-established risk factor for CVD (Paluch et al., 2024), urging greater focus on promoting physical activity (PA) through both aerobic and resistance training (RT) interventions (Lloyd-Jones et al., 2022). Data from a 20-year longitudinal study (Hyde et al., 2021) reveals that individuals engaging in aerobic programs are more likely to participate in RT (43.5%) compared to those who do not participate in aerobic programs (3.6%). These findings, together with results from a recent study by Cancela-Carral et al. (2025), underscore the pivotal role of maintaining high levels of PA, not only as a means to support participation in multimodal exercise routines like RT, but also as a comprehensive strategy for improving cardiovascular health, preserving functional independence, extending healthy lifespan, and enhancing overall quality of life in aging populations (Izquierdo et al., 2025; Lloyd-Jones et al., 2022).

Structured exercise programs have demonstrated effectiveness in mitigating age-related declines in ANS function, particularly in parasympathetic-related indices (Bhati et al., 2019; Grässler et al., 2021). However, in older populations, interruptions or discontinuation of community-based exercise programs are often unavoidable due to health, logistical, or seasonal factors (Douda et al., 2015). These disruptions may lead to a regression or complete loss of previously achieved autonomic benefits (Dias Reis et al., 2017; Douda et al., 2015; Heffernan et al., 2009). Despite this, research has largely overlooked PA behaviors following structured exercise programs, instead focusing on the detraining effects on cardiac autonomic control in various populations, including middle-aged and older adults (Ammar et al., 2021; Rodrigues et al., 2021), individuals with heart failure (Adamopoulos et al., 1995), cancer survivors (Dias Reis et al., 2017), young adults (Gamelin et al., 2007; Heffernan et al., 2007, 2009), and obese individuals (Park et al., 2019).

While these studies have provided valuable insights into detraining-induced changes in HRV, they may fall short in addressing an ethical challenge, specifically the lack of encouragement for participants to maintain PA levels or initiate new exercise programs after the exercise program cessation (Esmonde, 2023). Particularly, findings from Soares-Miranda et al. (2014) underscore that older adults should maintain at least modest PA levels as they age, highlighting the critical role of sustained lifestyle changes in promoting long-term cardiovascular and autonomic health. Therefore, to address this gap, the present study analyzed the influence of PA levels on the long-term retention of the effects on cardiac autonomic control, assessed by HRV, following a 16-week high-speed resistance training (HSRT) program over a 12-month follow-up period.

## Materials and Methods

### Study Design

This exploratory and longitudinal study belongs to the “*Active Aging*” research project, previously registered on *clinicaltrials.gov* (ID: NCT05586087). The project was applied in accordance with the Declaration of Helsinki and received ethical approval from the University of Evora Ethics Committee (clearance number: 22030). All participants were thoroughly informed of the study’s objectives, potential benefits, and risks, providing written informed consent before participation.

The present study exclusively included participants from the intervention group (IG) since participants from the control group (CG) were placed on a waiting list at pre-intervention in order to start new research projects after the 16-week intervention, making them ineligible for inclusion in this analysis. The results of the clinical trial design comparing the IG and CG over the 16-week HSRT program was published elsewhere.

The research committee overseeing the study did not approve the use of the term *detraining*, which traditionally involves a restriction on participants’ PA over a given period. Instead, the committee proposed encouraging IG participants to maintain their PA or start new exercise programs over the 12-month follow-up period.

### Participants

Following the intervention phase, a total of 37 older adults were available for the 12-month follow-up. However, one participant was lost to follow-up due to loss of contact, resulting in a final sample of 36 participants (mean age: 69.33 ± 3.12 years) included in the present analysis.

Based on their PA levels at 12-month follow-up, assessed using *the International Physical Activity Questionnaire-Short Form* (IPAQ-SF), participants were categorized into two groups: a light activity group (LAG; *N* = 20, mean age: 70.00 ± 3.66 years) and a moderate-to-vigorous activity group (MVAG; *N* = 16, mean age: 68.50 ± 2.09 years). Additionally, attrition was minimal (2.7%) and evenly distributed, with no evidence of differential dropout that could bias the results.

To be included in the study, participants had to meet the following inclusion criteria: (a) be at least 65 years old; (b) be able to walk independently; and (c) be capable of performing daily living tasks. Lastly, exclusion criteria included a diagnosis of diabetes or cardiac disease, recent surgery within the past 6 months, or an active oncological condition.

### Procedures

All assessments were scheduled in the morning between 8:30 a.m. and 10:30 a.m. Participants were instructed to arrive in a fast state, with an empty bladder, and have refrained from exercise, alcohol, and coffee consumption in the preceding 24 hr. The assessment order was as follows: anthropometric, blood pressure, and HRV. A single researcher performed all measurements in a consistent sequence for each participant.

### Measurements

#### Heart Rate Variability

The study adhered to the international guidelines for HRV measurements (Shaffer & Ginsberg, 2017; Task Force of the European Society of Cardiology and the North American Society of Pacing and Electrophysiology, 1996). The participants underwent 10-min dorsal decubitus stabilization before a 6-min recording assessment.

Sinus-origin heartbeat interval variation was gaged via a Polar^®^ H10 HR chest strap connected to the Elite HRV^®^ app (version 5.5.5, Elite^®^ HRV Inc., Asheville, USA) for recording and computation of HRV indices (Speer et al., 2020). The raw RR data, which were exported in a.*txt* file, were analyzed using the Kubios HRV software (version 3.5.0, University of Kuopio, Finland). Files were corrected for ectopic beats and artifacts using medium-level artifact correction (Moya-Ramon et al., 2022), with detailed algorithm descriptions in the Kubios software manual (Lipponen & Tarvainen, 2019).

For the time domain, following indices were computed: (i) mean RR intervals (mean RR) in milliseconds (ms); (ii) standard deviation of RR intervals (SDNN) in ms; (iii) root mean square of successive RR interval differences (RMSSD) in ms; (iv) percentage of successive RR intervals differing by >50 ms (pNN50); and (v) the stress index, representing the square root of Baevsky’s stress index (Baevsky & Berseneva, 2008).

For the frequency domain, the computed indices included: (i) LF, the absolute power of the low-frequency band (0.04–0.15 Hz) in ms^2^; (ii) HF, the absolute power of the high-frequency band (0.15–0.4 Hz) in ms^2^; (iii) the LF/HF ratio; and (iv) total power (TP) in ms^2^.

Lastly, the non-linear domain indices were: (i) SD1, the Poincaré plot standard deviation perpendicular to the line of identity in ms; (ii) SD2, the Poincaré plot standard deviation along the line of identity in ms; (iii) sample entropy (SampEn), indicating the probability of finding specific patterns in a short time series; and (iv) detrended fluctuation analysis (DFA α1), which describes short-term fluctuations.

#### Anthropometric and Blood Pressure

Anthropometric assessments included measuring weight and height by an electronic scale (TANITA^®^, MC 780MA, Amsterdam, Netherlands) and a stadiometer (SECA^®^ 220, Hamburg, Germany), respectively. Body mass index (BMI) was subsequently calculated via the standard formula: BMI = body mass (kg)/height² (m²).

Blood pressure was assessed using the Omron HEM-907 (Omron Healthcare Co. Ltd., Kyoto, Japan), with appropriately sized automated cuffs on the left arm. Two measurements, taken 1 min apart, were averaged after a 5-min rest period to ensure participant tranquility. The device provided readings for systolic blood pressure (SBP) and diastolic blood pressure (DBP). Afterward, pulse pressure (PP) was calculated by subtracting the DBP values from the SBP values.

#### Physical Activity

Participants’ PA levels were assessed using the IPAQ-SF (Craig et al., 2003; Grimm et al., 2012). This questionnaire gathered data on several metrics, including the number of activity days and minutes per week, as well as total PA, walking, moderate, and vigorous activity levels expressed in MET-minutes/week. The sitting time during weekdays and weekends were also recorded.

Based on the participants’ answers, they were then categorized into light, moderate, or vigorous activity levels (Supplemental Table A in the Supplemental File). The data were analyzed using a scoring spreadsheet developed by Cheng (2016). Lastly, Supplemental Table B in the Supplemental File summarizes the PA reported by participants at the 12-month follow-up.

### High-Speed Resistance Training Protocol

The HSRT program was performed three times weekly for 16 weeks under supervision. The prescription was adjusted every 2 weeks. Each session consisted of an initial warm-up (10–15 min), the main HSRT exercises (45–55 min), and a concluding cool-down phase (5–10 min). The main phase included the following upper- and lower-body exercises: squats on smith machine or with dumbbells (depending on each participant’s ability); leg press, leg extension; calf raise; seated row; peck fly; lat pull down; and incline bench press (Technogym, SPA, Cesena, Italy).

This present training protocol employs progressively increasing loads, tailored to the participants’ mean concentric phase velocity for each set across all exercises (B. Mann, 2016; J. B. Mann et al., 2015): 1st to 4th weeks, an average speed over 1.3 m/s was required (*starting strength*); from the 5th to 10th weeks, speeds were adjusted to between 1.3 and 1.0 m/s (*speed/strength*); and in the 11th to 16th weeks, speeds ranged from 1.0 to 0.75 m/s (*strength/speed*). The mean concentric phase velocity for each set and exercise was monitored using a BEAST™ sensor (Beast Technologies, Brescia, Italy) (Vallejo et al., 2020). This device provided real-time feedback on instantaneous velocity to both participants and supervisors and displayed the mean velocity at the end of each set. Each session used six accelerometers connected via Bluetooth to six separate cell phones. Participants were also actively encouraged to execute each repetition swiftly and explosively, while maintaining a controlled pace of 2 to 3 s during the eccentric phase.

The intensity of the exercises was evaluated using the rating of perceived exertion (RPE) scale, developed by Borg (1982), and heart rate monitoring (Polar M200, Polar Electro Ltd). The training period passed without any adverse events, and participant attendance was diligently recorded.

### Statistical Analysis

Prior to the study, a sample size calculation was performed via G-power software (University of Dusseldorf, Germany; Faul et al., 2009) for *F* tests through ANOVA, repeated measured, within factors: *f* = 0.25, α error probability = .05, power (1-β err prob) = .80, number of groups = 1, and number of measurements = 4. The actual power output indicated that this clinical trial should include at least 24 participants to achieve an 82% chance of successfully rejecting the null hypothesis. All statistical analyses were conducted using SPSS for Windows, version 26 (IBM Corp., Armonk, NY, USA), with the significance level set at *p* ≤ .050 (two-tailed). To complement traditional null hypothesis significance testing, an estimation approach was employed (Cumming & Calin-Jageman, 2017; Ho et al., 2019).

To evaluate the time effect across the four measurement points for each outcome, repeated measures ANOVA were applied. Pairwise comparisons between time points and groups were conducted using the Bonferroni post-hoc test. The effect sizes (ESs) were then calculated according to Cohen (1988), using Cohen’s *d_unbiased_* (*d_unb_*) through a specific spreadsheet (Cumming & Calin-Jageman, 2017). While the ESs for ANOVA were expressed as partial eta-squared values (η_p_^2^) and interpreted using the following thresholds: 0.010 to 0.059 (small), 0.060 to 0.140 (medium), and greater than 0.140 (large), the pairwise comparisons’ ESs, expressed as *d_unb_*, were categorized as: less than 0.20 (trivial), 0.20 to 0.49 (small), 0.50 to 0.80 (medium), and greater than 0.80 (large). Finally, graphical representations were created using the RStudio software.

## Results

### Participants

The participants’ general characteristics included in the present study are shown in the Supplemental File as Supplemental Table C. Several significant differences were observed in age, weight, and BMI over the study period.

### Heart Rate Variability

Table 1 shows the changes in HRV indices across the four measurement points. Among these, only DFA α1 demonstrated a significant time effect, with a moderate ES, throughout the study period.

**Table 1.** Changes in Heart Rate Variability Indices Over the Study Period.

Measures	Groups	Intervention		Follow-up		Time effect	Interaction effect within groups	Interaction effect between groups
		M0 - Pre	M1- Post	M2 - 6-month	M3 - 12-month			
General								
SBP (mmHg)	LAG	134.50 ± 18.24	130.00 ± 11.04	128.10 ± 17.96	128.05 ± 13.25	*F* = 4.988 *p* ** = .003** η²_p_ = 0.128^*^	*F* = 0.564 *p* = .640 η²_p_ = 0.016^#^	*F* = 1.349 *p* = .254 η²_p_ = 0.038^#^
	MVAG^a^	134.38 ± 11.46	124.13 ± 13.85	121.25 ± 16.75	123.38 ± 16.82			
DBP (mmHg)	LAG^b^	79.85 ± 10.86	82.65 ± 8.92	77.90 ± 10.94	77.15 ± 7.09	*F* = 3.444 *p* ** = .020** η²_p_ = 0.092*	*F* = 1.650 *p* = .182 η²_p_ = 0.046^#^	*F* = 0.217 *p* = .644 η²_p_ = 0.006
	MVAG	82.38 ± 10.24	77.81 ± 8.29	77.00 ± 7.72	76.00 ± 8.63			
PP (mmHg)	LAG	54.65 ± 13.99	47.35 ± 7.14	50.20 ± 10.57	50.90 ± 10.65	*F* = 3.204^¥^ *p* ** = .037** η²_p_ = 0.086*	*F* = 0.381^¥^ *p* = .721 η²_p_ = 0.011^#^	*F* = 1.791 *p* = .190 η²_p_ = 0.050^#^
	MVAG	52.00 ± 10.67	46.31 ± 10.45	44.25 ± 11.40	47.38 ± 14.25			
Mean HR (bpm)	LAG	61.25 ± 11.63	58.60 ± 6.75	60.15 ± 6.72	60.65 ± 6.54	*F* = 2.126^¥^ *p* = .143 η²_p_ = 0.059^#^	*F* = 1.328^¥^ *p* = .268 η²_p_ = 0.038^#^	*F* = 2.021 *p* = .164 η²_p_ = 0.056^#^
	MVAG^c^	66.38 ± 11.11	62.75 ± 7.91	63.38 ± 8.33	60.19 ± 6.04			
Min HR (bpm)	LAG	55.70 ± 7.85	54.70 ± 7.09	55.85 ± 6.15	56.70 ± 6.59	*F* = 1.574^¥^ *p* = .216 η²_p_ = 0.044^#^	*F* = 2.452^¥^ *p* = .098 η²_p_ = 0.067*	*F* = 1.077 *p* = .307 η²_p_ = 0.031^#^
	MVAG^c^	60.75 ± 11.08	57.31 ± 8.31	58.44 ± 8.94	55.38 ± 5.28			
Max HR (bpm)	LAG	64.35 ± 7.01^†^	63.40 ± 6.69^†^	66.00 ± 7.83	66.10 ± 7.48	*F* = 2.144^¥^ *p* = .131 η²_p_ = 0.059*	*F* = 3.297^¥^ *p* ** = .048** η²_p_ = 0.088*	*F* = 9.124 *p* ** = .005** η²_p_ = 0.212^§^
	MVAG	76.13 ± 13.68	69.56 ± 8.64	71.00 ± 7.76	67.56 ± 8.66			
Time-domain								
Mean RR (ms)	LAG	1004.20 ± 146.13	1036.30 ± 125.75	1009.95 ± 111.96	999.75 ± 105.32	*F* = 1.575^¥^ *p* = .216 η²_p_ = 0.044^#^	*F* = 1.623^¥^ *p* = .206 η²_p_ = 0.046^#^	*F* = 2.271 *p* = .141 η²_p_ = 0.063*
	MVAG^c^	928.00 ± 161.82	970.44 ± 109.88	944.19 ± 142.83	1005.94 ± 91.85			
SDNN (ms)	LAG	28.63 ± 26.24	27.55 ± 14.67	24.84 ± 8.55	21.45 ± 8.16	*F* = 0.600^¥^ *p* = .551 η²_p_ = 0.017^#^	*F* = 0.372^¥^ *p* = .690 η²_p_ = 0.011^#^	*F* = 1.049 *p* = .313 η²_p_ = 0.030^#^
	MVAG	38.26 ± 42.66	35.79 ± 52.58	36.91 ± 58.78	36.41 ± 41.63			
RMSSD (ms)	LAG	30.65 ± 20.59	32.96 ± 22.58	28.13 ± 12.28	22.82 ± 8.33	*F* = 0.573^¥^ *p* = .590 η²_p_ = 0.017^#^	*F* = 2.145^¥^ *p* = .117 η²_p_ = 0.059^#^	*F* = 0.011 *p* = .918 η²_p_ = 0.001
	MVAG	31.25 ± 17.21	26.68 ± 20.09	24.54 ± 18.13	34.05 ± 31.80			
pNN50 (%)	LAG	7.50 ± 15.46	9.09 ± 12.32	5.55 ± 6.99	4.35 ± 4.70	*F* = 2.298^¥^ *p* = .101 η²_p_ = 0.063^*^	*F* = 0.328^¥^ *p* = .747 η²_p_ = 0.010^#^	*F* = 0.013 *p* = .909 η²_p_ = 0.001
	MVAG	9.74 ± 19.18	9.14 ± 22.49	3.64 ± 6.37	5.61 ± 10.23			
Stress index	LAG	14.75 ± 5.74	13.57 ± 5.86	13.98 ± 4.45	16.51 ± 5.18	*F* = 0.985 *p* = .403 η²_p_ = 0.028^#^	*F* = 3.277 *p* ** = .024** η²_p_ = 0.088*	*F* = 0.026 *p* = .873 η²_p_ = 0.001
	MVAG	12.08 ± 5.25	16.00 ± 8.62	16.05 ± 7.35	13.72 ± 5.50			
Frequency domain								
LF (ms^2^)	LAG	294.50 ± 282.01	316.05 ± 266.23	321.90 ± 275.57	225.40 ± 225.98	*F* = 0.169 *p* = .917 η²_p_ = 0.005	*F* = 0.755 *p* = .522 η²_p_ = 0.022^#^	*F* = 0.289 *p* = .595 η²_p_ = 0.008
	MVAG	323.56 ± 280.65	292.13 ± 267.02	333.38 ± 352.09	354.63 ± 301.11			
LF (nu)	LAG	48.02 ± 15.19	48.33 ± 18.20	53.65 ± 20.66	53.89 ± 17.26	*F* = 1.693 *p* = .173 η²_p_ = 0.047^#^	*F* = 0.163 *p* = .921 η²_p_ = 0.005	*F* = 1.284 *p* = .265 η²_p_ = 0.036^#^
	MVAG	54.62 ± 19.34	53.26 ± 26.73	61.28 ± 18.51	56.95 ± 19.10			
HF (ms^2^)	LAG	221.35 ± 195.42	307.85 ± 264.07	259.40 ± 220.83	165.30 ± 124.58	*F* = 0.763^¥^ *p* = .495 η²_p_ = 0.022^#^	*F* = 2.030^¥^ *p* = .127 η²_p_ = 0.056^#^	*F* = 0.945 *p* = .338 η²_p_ = 0.027^#^
	MVAG	269.38 ± 256.00	327.44 ± 334.37	244.44 ± 307.09	390.50 ± 512.09			
HF (nu)	LAG	51.93 ± 15.18	51.57 ± 18.11	46.26 ± 20.58	46.04 ± 17.16	*F* = 1.686 *p* = .175 η²_p_ = 0.047^#^	*F* = 0.167 *p* = .918 η²_p_ = 0.005	*F* = 1.294 *p* = .263 η²_p_ = 0.037^#^
	MVAG	45.22 ± 19.10	46.69 ± 26.71	38.67 ± 18.50	43.01 ± 19.07			
Ratio LF/HF	LAG	1.14 ± 0.85	1.25 ± 1.01	1.61 ± 1.39	1.46 ± 1.05	*F* = 1.243 *p* = .298 η²_p_ = 0.035^#^	*F* = 0.389 *p* = .761 η²_p_ = 0.011^#^	*F* = 2.319 *p* = .137 η²_p_ = 0.064*
	MVAG	1.59 ± 1.06	2.02 ± 1.91	1.95 ± 1.23	1.83 ± 1.31			
TP (ms^2^)	LAG	408.15 ± 273.00	541.90 ± 364.53	630.45 ± 424.52	449.60 ± 344.81	*F* = 2.050^¥^ *p* = .125 η²_p_ = 0.057^#^	*F* = 2.305^¥^ *p* = .095 η²_p_ = 0.064*	*F* = 0.932 *p* = .341 η²_p_ = 0.027^#^
	MVAG	495.06 ± 355.19	530.19 ± 358.95	618.00 ± 635.04	824.88 ± 785.39			
Non-linear domain								
SD1 (ms)	LAG	23.16 ± 20.15	24.46 ± 16.09	20.54 ± 9.45	16.11 ± 5.89	*F* = 0.631^¥^ *p* = .547 η²_p_ = 0.018^#^	*F* = 2.021^¥^ *p* = .136 η²_p_ = 0.056^#^	*F* = 0.001 *p* = .996 η²_p_ = 0.001
	MVAG	22.37 ± 12.06	19.47 ± 15.73	18.24 ± 13.43	24.11 ± 22.53			
SD2 (ms)	LAG	29.55 ± 18.24	29.73 ± 14.60	28.61 ± 10.47	25.37 ± 10.79	*F* = 0.272^¥^ *p* = .768 η²_p_ = 0.008	*F* = 0.412^¥^ *p* = .669 η²_p_ = 0.012^#^	*F* = 1.780 *p* = .191 η²_p_ = 0.050^#^
	MVAG	35.17 ± 18.85	31.66 ± 17.98	32.13 ± 18.07	33.88 ± 16.98			
SampEn	LAG	1.80 ± 0.31^†^	1.72 ± 0.30	1.69 ± 0.31	1.82 ± 0.21^†^	*F* = 0.560 *p* = .623 η²_p_ = 0.016^#^	*F* = 1.887 *p* = .137 η²_p_ = 0.053^#^	*F* = 7.527 *p* **= .010** η²_p_ = 0.181^§^
	MVAG	1.43 ± 0.40	1.59 ± 0.34	1.54 ± 0.37	1.54 ± 0.38			
DFA α1	LAG	0.85 ± 0.16	0.84 ± 0.29	0.89 ± 0.25^†^	0.95 ± 0.25	*F* = 3.546 *p* **= .017** η²_p_ = 0.094*	*F* = 1.164 *p* = .327 η²_p_ = 0.033^#^	*F* = 2.689 *p* = .110 η²_p_ = 0.073*
	MVAG	0.89 ± 0.30	0.99 ± 0.37	1.10 ± 0.26	1.02 ± 0.26			

*Note.* Significant differences between periods are highlighted in bold (p ≤ .050). LAG = light activity group; MVAG = moderate-to-vigorous activity group; SBP = systolic blood pressure; DBP = diastolic blood pressure; PP = pulse pressure; mmHg = millimeter of mercury; HR = heart rate; bpm = beats per minute; ms = milliseconds; SDNN = standard deviation of RR; RMSSD; root mean square of successive RR interval differences; pNN50 = percentage of successive RR intervals differing by >50 ms; LF = low frequency; HF = high frequency; nu = normalized units; TP = total power; SampEn = sample entropy; DFA = detrended fluctuation analysis.

¥ Greenhouse−Geisser correction.

Significant differences:

a Pre-intervention versus 6-month follow-up.

b Post-intervention versus 12-month follow-up.

c Six-month follow-up versus 12-month follow-up.

† Between groups at that assessment point.

η²_p_ values thresholds:

# Small effect: 0.010 to 0.059;

* Medium effect: 0.060 to 0.140;

§ Large effect: >0.140.

Table 1 also exhibits the interaction effects within and between groups. In MVAG, the SBP was significantly higher at pre-intervention compared to the 6-month follow-up (*p* = .014, *d*_unb_ = −0.87 [−1.43 to −0.38]), while in LAG, the DBP was significantly higher at post-intervention than 12-month follow-up (*p* = .037, *d*_unb_ = −0.66 [−1.14 to −0.22]). Moreover, Min and Mean HR values were significantly higher at the 6-month compared to the 12-month follow-up (*p* = .045, *d*_unb_ = −0.39 [−0.80 to −0.02] and *p* = .037, *d*_unb_ = −0.42 [−0.83 to −0.03], respectively) in the MVAG. For Max HR, the LAG exhibited significantly lower values than the MVAG at pre- (*p* = .002, *d*_unb_ = 1.09 [0.41–1.83]) and post-intervention (*p* = .021, *d*_unb_ = 0.79 [0.12–1.49]). In addition, the MVAG demonstrated significantly higher Mean RR values at the 12-month compared to the 6-month follow-up (*p* = .043, *d*_unb_ = 0.49 [0.01–1.01]).

Furthermore, SampEn was significantly higher in the LAG compared to the MVAG at pre-intervention (*p* = .003, *d*_unb_ = −1.03 [−1.75 to −0.35]) and 12-month follow-up (*p* = .010 *d*_unb_ = −0.89 [−1.61 to −0.22]). Lastly, the LAG also showed significantly lower DFA α1 values compared to the MVAG at 6-month follow-up (*p* = .025, *d*_unb_ = 0.77 [0.09–1.46]). Variations in HRV indices over the study period are further illustrated in Supplemental Figure S1 of the Supplemental File.

## Discussion

This study investigated whether different levels of PA (light vs. moderate-to-vigorous activity) influenced of the effects on cardiac autonomic control, assessed by HRV, following a 16-week HSRT program over a 12-month follow-up period. The main study’s findings were: (i) MVAG demonstrated better values in parasympathetic-related indices, including RMSSD, HF (ms^2^), and SD1; (ii) the MVAG also exhibited higher values in indices representing overall ANS function, such as SDNN, TP, and SD2; (iii) the LAG showed poorer results for stress index and SampEn at 12-month follow-up period; and (iv) higher PA levels appear to play an important role to blood pressure regulation. In summary, the ANS modulation achieved during the intervention was mostly and gradually reversed over the follow-up among LAG participants.

Unlike previous studies on HRV detraining effects after structured exercise programs, including RT (Heffernan et al., 2007, 2009), aerobic (Adamopoulos et al., 1995; Ammar et al., 2021; Gamelin et al., 2007; Park et al., 2019), combined (Dias Reis et al., 2017), respiratory (Rodrigues et al., 2021), and yoga (Uthiravelu et al., 2023), the present study encouraged participants to maintain or increase PA levels post-intervention. This approach shifts the focus from detraining effects to importance of sustained lifestyle habits for long-term cardiovascular and ANS health (Soares-Miranda et al., 2014).

The study findings revealed that MVAG participants exhibited higher values in parasympathetic-related indices, including RMSSD, pNN50%, HF (ms²), and SD1. In contrast, the LAG participants showed declines in these indices over time (Table 1). Although no significant differences were observed between groups or time points, LAG demonstrated a 31% downward trend in RMSSD (*medium* ES), while the MVAG revealed a 28% upward trend (*small* ES) from post-intervention to 12-month follow-up. Similar patterns were observed for pNN50, HF (ms^2^), and SD2. Prior studies also reported declines in RMSSD after intervention cessation. For instance, a 6-week RT intervention in young men followed by 4-week detraining period showed RMSSD reductions (Heffernan et al., 2009). Similarly, Adamopoulos et al. (1995) found significant RMSSD and pNN50 reductions during an 8-week detraining period after an aerobic intervention in chronic heart failure patients. These findings align with earlier studies highlighting parasympathetic declines post-intervention (Ammar et al., 2021; Dias Reis et al., 2017; Gamelin et al., 2007; Heffernan et al., 2009; Uthiravelu et al., 2023).

A plausible explanation for the decline in vagal activity observed in the LAG may involve a gradual reduction in baroreflex homeostasis over time (Selig et al., 2004). Additionally, possible vascular adaptations induced by the 16-week HSRT, such as improved forearm blood flow (Selig et al., 2004) and increased nitric oxide bioavailability (Macedo et al., 2016), might have reverted to pre-intervention levels, particularly among participants who did not engage in regular PA (seven participants). These findings underscore the role of sustained PA as a protective factor against ANS imbalance, supporting parasympathetic activity and mitigating the age-related decline previously observed in this population (Soares-Miranda et al., 2014). Nonetheless, future studies are needed to confirm such hypothetical adaptations.

SDNN is a well-established indicator of overall ANS function (Shaffer & Ginsberg, 2017; Task Force of the European Society of Cardiology and the North American Society of Pacing and Electrophysiology, 1996). In short-term resting assessments, its primary source of variation is parasympathetically mediated respiratory sinus arrhythmia (Shaffer et al., 2014). In the present study, opposite patterns were observed for both groups (Table 1). The LAG exhibited a 22% decrease from post-intervention to the 12-month follow-up (*small ES*), whereas the MVAG preserved their post-intervention values within an acceptable range. This retention aligns with a previous research following the cessation of RT (Heffernan et al., 2007). Additionally, after the HSRT program, the LAG exhibited a 17% reduction in TP values (*small ES*) at the 12-month follow-up, while the MVAG presented a 56% increase (*small ES*). A *moderate* ES for the interaction between groups supports these results, aligning with findings from previous studies with shorter follow-up periods (Adamopoulos et al., 1995; Uthiravelu et al., 2023). As TP reflects the group dynamics of overall ANS function (Shaffer & Ginsberg, 2017), its increase may be attributed to a rise in HF component. The sympathetic activation typically induces tachycardia and a pronounced reduction in TP, whereas vagal activation leads in the opposite effect (Task Force of the European Society of Cardiology and the North American Society of Pacing and Electrophysiology, 1996).

Regarding sympathetic activity, DFA α1 stabilized near a value of one in both groups after HSRT (Table 1). Despite a significant group difference at the 6-month follow-up, with higher values observed in the MVAG compared to the LAG (*medium* ES), both groups displayed higher values at the 12-month follow-up than pre-intervention. This finding is relevant, as values near one (indicative of pink noise), reflect an optimal balance that helps biological systems avoiding excessive order or chaos (Stergiou, 2020). On the other hand, a loss of fractal complexity compromises control systems and reduces adaptability (Harrison & Stergiou, 2015).

Similarly, the complexity measure SampEn exhibited significantly higher values in the LAG at the 12-month follow-up (*large* ES). According to Stergiou (2020), higher SampEn values suggest more random or chaotic behavior. Even though the significant differences at baseline could have influenced the results, the observed changes warrant attention by exercise and health professionals. Previously, two studies on young populations (Heffernan et al., 2007, 2009) reported similar patterns, with post-intervention values returning to baseline. Potential mechanisms (i.e., multiple attractors) could have contributed to these results, such as the baroreflex was reset to a new pressure operating range after HSRT (Tatro et al., 1992), thus augmenting cardiac vagal outflow and the releasing of high doses of atropine can reduce heart complexity (Porta et al., 2007). However, limited research has examined post-intervention patterns of DFA α1 and SampEn. Given their promising potential as indicators of physiological adaptations (Stergiou, 2020), future studies should incorporate them to deepen our understanding.

Finally, the blood pressure results demonstrated beneficial reductions in both groups. Specifically, the MVAG showed a significant decrease in SBP from pre-intervention to the 6-month follow-up (*large* ES), with a cumulative 8% reduction by 12 months. Both groups also experienced meaningful reductions in PP, with declines of 6% in LAG and 8% in MVAG across the study period (M0–M3). These findings align with Cancela-Carral et al. (2025), who reported that for older adults with hypertension, 60 min of aerobic or RT twice a week is adequate, with RT providing greater advantages. The observed improvements may be attributed to reduced arterial stiffness, heightening the clinical relevance of PA habits in mitigating cardiovascular risk factors and reducing the likelihood of cardiovascular events in this population (Kannel, 2000).

Nonetheless, some limitations of this study should be acknowledged. First, although a priori power analysis indicated that a sample of 24 participants would be sufficient to detect meaningful effects with 82% power, the final sample sizes in subgroup analyses may limit the generalizability of the findings and increase the risk of Type II errors. Second, although the 6-min HRV recording duration followed established international HRV standards, it may not fully capture diurnal variations or provide the depth information available from long-term recordings. Future studies should consider using 24-hr Holter monitoring to more comprehensively assess ANS modulation across different times of day and activity contexts. Third, the assessor’s awareness of the study objectives could have introduced bias due to expectancy effects. Second, while participants were instructed to maintain normal breathing during the assessments, the number of breathing cycles was not recorded. Lastly, PA levels were assessed using a self-reported, indirect tool, which may have introduced inaccuracies in estimating individual PA behavior.

## Conclusion

This study highlights that maintaining at least moderate levels of PA (i.e., an active lifestyle) following a HSRT program is important for sustaining the HRV benefits achieved in older adults. Specifically, participants in the MVAG demonstrated better retention of parasympathetic-related indices, including RMSSD, HF (ms²), and SD1, and overall ANS markers, such as SDNN and TP. Furthermore, the results suggest that non-linear HRV indices such as DFA α1 and SampEn may offer valuable insights into autonomic adaptability and should be explored further in future research.

Overall, these findings reinforce the key role of sustained PA in promoting long-term cardiovascular and autonomic health in older adults. Encouraging active lifestyles beyond structured programs may be key to maximizing and maintaining physiological adaptations during aging.

## Supplemental Material

sj-docx-1-ggm-10.1177_30495334251369446 – Supplemental material for Long-Term Impact of Physical Activity Levels After High-Speed Resistance Training on Cardiac Autonomic Control in Independent Older Adults Supplemental material, sj-docx-1-ggm-10.1177_30495334251369446 for Long-Term Impact of Physical Activity Levels After High-Speed Resistance Training on Cardiac Autonomic Control in Independent Older Adults by Alexandre Duarte Martins, Orlando Fernandes, João Paulo Brito, Rafael Oliveira, Bruno Gonçalves and Nuno Batalha in Sage Open Aging

## References

[bibr1-30495334251369446] Adamopoulos S. Ponikowski P. Cerquetani E. Piepoli M. Rosano G. Sleight P. Coats A. J. (1995). Circadian pattern of heart rate variability in chronic heart failure patients. Effects of physical training. European Heart Journal, 16(10), 1380–1386. 10.1093/oxfordjournals.eurheartj.a060746 8746907

[bibr2-30495334251369446] Ammar A. Boukhris O. Halfpaap N. Labott B. K. Langhans C. Herold F. Grässler B. Müller P. Trabelsi K. Chtourou H. Zmijewski P. Driss T. Glenn J. M. Müller N. G. Hoekelmann A. (2021). Four weeks of detraining induced by COVID-19 reverse cardiac improvements from eight weeks of fitness-dance training in older adults with mild cognitive impairment. International Journal of Environmental Research and Public Health, 18(11), 5930. 10.3390/ijerph18115930 34073051 PMC8198940

[bibr3-30495334251369446] Baevsky R. M. Berseneva A. P. (2008). Methodical recommendations use kardivar system for determination of the stress level and estimation of the body adaptability standards of measurements and physiological interpretation. https://www.academia.edu/35296847/Methodical_recommendations_USE_KARDiVAR_SYSTEM_FOR_DETERMINATION_OF_THE_STRESS_LEVEL_AND_ESTIMATION_OF_THE_BODY_ADAPTABILITY_Standards_of_measurements_and_physiological_interpretation_Moscow_Prague_2008

[bibr4-30495334251369446] Bhati P. Moiz J. A. Menon G. R. Hussain M. E. (2019). Does resistance training modulate cardiac autonomic control? A systematic review and meta-analysis. Clinical Autonomic Research, 29(1), 75–103. 10.1007/s10286-018-0558-3 30141031

[bibr5-30495334251369446] Borg G. (1982). Phychophysical bases of perceived exertion. Medicine and Science in Sports and Exercise, 14(5), 377–381. 10.1249/00005768-198205000-00012 7154893

[bibr6-30495334251369446] Cancela-Carral J. M. Bezerra P. Lopez-Rodriguez A. Silva B. (2025). Differential effects of the type of physical exercise on blood pressure in independent older adults. Sports Health. A﻿dvance online publication. 10.1177/19417381241303706 PMC11699555 39754304

[bibr7-30495334251369446] Cheng H. L. (2016). A simple, easy-to-use spreadsheet for automatic scoring of the International Physical Activity Questionnaire (IPAQ) Short Form (updated November 2016). ResearchGate. https://tinyurl.com/24hzlxcg

[bibr8-30495334251369446] Cohen J. (Ed.). (1988). Statistical power analysis for the behavioral sciences (2nd ed.). Lawrence Erlbaum Associates.

[bibr9-30495334251369446] Craig C. L. Marshall A. L. Sjöström M. Bauman A. E. Booth M. L. Ainsworth B. E. Pratt M. Ekelund U. Yngve A. Sallis J. F. Oja P. (2003). International Physical Activity Questionnaire: 12-Country Reliability and validity. Medicine and Science in Sports and Exercise, 35(8), 1381–1395. 10.1249/01.MSS.0000078924.61453.FB 12900694

[bibr10-30495334251369446] Cumming G. Calin-Jageman R. (Eds.). (2017). Introduction to the New Statistics: Estimation, Open Science, and Beyond (1st ed.). Routledge. https://www.esci.thenewstatistics.com

[bibr11-30495334251369446] Dias Reis A. João B. Garcia S. Rodrigues Diniz R. Silva-Filho A. C. Dias C. J. Leite R. D. Mostarda C . (2017). Effect of exercise training and detraining in autonomic modulation and cardiorespiratory fitness in breast cancer survivors. Journal of Sports Medicine and Physical Fitness, 57, 1062–1068. 10.23736/S0022-4707.17.07012-8 28134506

[bibr12-30495334251369446] Díez-Villanueva P. Jiménez-Méndez C. Bonanad C. García-Blas S. Pérez-Rivera Allo G García-Pardo H Formiga F Camafort M Martínez-Sellés M Ariza-Solé A Ayesta A. (2022). Risk factors and cardiovascular disease in the elderly. Reviews in Cardiovascular Medicine, 23(6), 188. 10.31083/j.rcm2306188 39077174 PMC11273864

[bibr13-30495334251369446] Douda H. T. Kosmidou K. V. Smilios I. Volaklis K. A. Tokmakidis S. P. (2015). Community-based training-detraining intervention in older women: A five-year follow-up study. Journal of Aging and Physical Activity, 23(4), 496–512. 10.1123/japa.2013-0241 25415933

[bibr14-30495334251369446] Esmonde K. (2023). Exercising caution: A case for ethics analysis in physical activity promotion. Public Health Ethics, 16(1), 77–85. 10.1093/phe/phad004

[bibr15-30495334251369446] Fang S.-C. Wu Y.-L. Tsai P.-S. (2020). Heart rate variability and risk of all-cause death and cardiovascular events in patients with cardiovascular disease: A meta-analysis of cohort studies. Biological Research for Nursing, 22(1), 45–56. 10.1177/1099800419877442 31558032

[bibr16-30495334251369446] Faul F. Erdfelder E. Buchner A. Lang A.-G. (2009). Statistical power analyses using G*Power 3.1: Tests for correlation and regression analyses. Behavior Research Methods, 41(4), 1149–1160. 10.3758/BRM.41.4.1149 19897823

[bibr17-30495334251369446] Freitas V. P. D. Passos R. D. S. Oliveira A. A. Ribeiro J. S. Freire I. V. Schettino L. Teles M. F. Casotti C. A. Pereira R. (2018). Sarcopenia is associated to an impaired autonomic heart rate modulation in community-dwelling old adults. Archives of Gerontology and Geriatrics, 76, 120–124. 10.1016/j.archger.2018.01.006 29494872

[bibr18-30495334251369446] Gamelin F. X. Berthoin S. Sayah H. Libersa C. Bosquet L. (2007). Effect of training and detraining on heart rate variability in healthy young men. International Journal of Sports Medicine, 28(7), 564–570. 10.1055/s-2007-964861 17373601

[bibr19-30495334251369446] Grässler B. Thielmann B. Böckelmann I. Hökelmann A. (2021). Effects of different exercise interventions on heart rate variability and cardiovascular health factors in older adults: A systematic review. European Review of Aging and Physical Activity, 18(1), 24. 10.1186/s11556-021-00278-6 34789148 PMC8597177

[bibr20-30495334251369446] Grimm E. K. Swartz A. M. Hart T. Miller N. E. Strath S. J. (2012). Comparison of the IPAQ-Short form and accelerometry predictions of physical activity in older adults. Journal of Aging and Physical Activity, 20(1), 64–79. 10.1123/japa.20.1.64 22190120

[bibr21-30495334251369446] Harrison S. J. Stergiou N. (2015). Complex adaptive behavior and dexterous action. Nonlinear Dynamics Psychology and Life Sciences, 19(4), 345–394. 26375932 PMC4755319

[bibr22-30495334251369446] Heffernan K. S. Fahs C. A. Shinsako K. K. Jae S. Y. Fernhall B. (2007). Heart rate recovery and heart rate complexity following resistance exercise training and detraining in young men. American Journal of Physiology-Heart and Circulatory Physiology, 293(5), H3180–H3186. 10.1152/ajpheart.00648.2007 17890428

[bibr23-30495334251369446] Heffernan K. S. Jae S. Y. Vieira V. J. Iwamoto G. A. Wilund K. R. Woods J. A. Fernhall B. (2009). C-reactive protein and cardiac vagal activity following resistance exercise training in young African-American and white men. American Journal of Physiology. Regulatory, Integrative and Comparative Physiology, 296(4), R1098–R1105. 10.1152/ajpregu.90936.2008 19193941

[bibr24-30495334251369446] Ho J. Tumkaya T. Aryal S. Choi H. Claridge-Chang A. (2019). Moving beyond P values: Data analysis with estimation graphics. Nature Methods, 16(7), 565–566. 10.1038/s41592-019-0470-3 31217592

[bibr25-30495334251369446] Hyde E. T. Whitfield G. P. Omura J. D. Fulton J. E. Carlson S. A. (2021). Trends in meeting the physical activity guidelines: Muscle-strengthening alone and combined with aerobic activity, United States, 1998-2018. Journal of Physical Activity and Health, 18(S1), S37–S44. 10.1123/jpah.2021-0077 PMC11000248 34465652

[bibr26-30495334251369446] Izquierdo M. de Souto Barreto P. Arai H. Bischoff-Ferrari H. A. Cadore E. L. Cesari M. Chen L.-K. Coen P. M. Courneya K. S. Duque G. Ferrucci L. Fielding R. A. García-Hermoso A. Gutiérrez-Robledo L. M. Harridge S. D. Kirk B. Kritchevsky S. Landi F. Lazarus N. . . . Fiatarone Singh M. A. (2025). Global consensus on optimal exercise recommendations for enhancing healthy longevity in older adults (ICFSR). Journal of Nutrition Health & Aging, 29(1), 100401. 10.1016/j.jnha.2024.100401 PMC11812118 39743381

[bibr27-30495334251369446] Kannel W. B. (2000). Elevated systolic blood pressure as a cardiovascular risk factor. American Journal of Cardiology, 85(2), 251–255. 10.1016/s0002-9149(99)00635-9 10955386

[bibr28-30495334251369446] Lipponen J. A. Tarvainen M. P. (2019). A robust algorithm for heart rate variability time series artefact correction using novel beat classification. Journal of Medical Engineering & Technology, 43(3), 173–181. 10.1080/03091902.2019.1640306 31314618

[bibr29-30495334251369446] Lloyd-Jones D. M. Allen N. B. Anderson C. A. M. Black T. Brewer L. C. Foraker R. E. Grandner M. A. Lavretsky H. Perak A. M. Sharma G. Rosamond W. (2022). Life’s essential 8: Updating and enhancing the American Heart Association’s construct of Cardiovascular Health: A Presidential Advisory from the American Heart Association. Circulation, 146(5), e18–e43. 10.1161/CIR.0000000000001078 PMC10503546 35766027

[bibr30-30495334251369446] Macedo F. N. Mesquita T. R. Melo V. U. Mota M. M. Silva T. L. Santana M. N. Oliveira L. R. Santos R. V. Miguel Dos Santos R. Lauton-Santos S. Santos M. R. Barreto A. S. Santana-Filho V. J. (2016). Increased nitric oxide bioavailability and decreased sympathetic modulation are involved in vascular adjustments induced by low-intensity resistance training. Frontiers in Physiology, 7, 265. 10.3389/fphys.2016.00265 27445854 PMC4923192

[bibr31-30495334251369446] Mann B. (2016). Developing explosive athletes: Use of velocity based training in athletes (3rd ed.). Ultimate Athlete Concepts.

[bibr32-30495334251369446] Mann J. B. Ivey P. A. Sayers S. P. (2015). Velocity-based training in football. Strength and Conditioning Journal, 37(6), 52–57. 10.1519/ssc.0000000000000177

[bibr33-30495334251369446] Moya-Ramon M. Mateo-March M. Peña-González I. Zabala M. Javaloyes A. (2022). Validity and reliability of different smartphones applications to measure HRV during short and ultra-short measurements in elite athletes. Computer Methods and Programs in Biomedicine, 217, 106696. 10.1016/j.cmpb.2022.106696 35172251

[bibr34-30495334251369446] Nowak K. L. Rossman M. J. Chonchol M. Seals D. R. (2018). Strategies for achieving healthy vascular aging. Hypertension, 71(3), 389–402. 10.1161/hypertensionaha.117.10439 29311256 PMC5812814

[bibr35-30495334251369446] Ogliari G. Mahinrad S. Stott D. J. Jukema J. W. Mooijaart S. P. Macfarlane P. W. Clark E. N. Kearney P. M. Westendorp R. G. J. de Craen A. J. M. Sabayan B. (2015). Resting heart rate, heart rate variability and functional decline in old age. Canadian Medical Association Journal, 187(15), E442–E449. 10.1503/cmaj.150462 PMC4610849 26323697

[bibr36-30495334251369446] Paluch A. E. Boyer W. R. Franklin B. A. Laddu D. Lobelo F. Lee D. C. McDermott M. M. Swift D. L. Webel A. R. Lane A. (2024). Resistance exercise training in individuals with and without cardiovascular disease: 2023 update: A Scientific Statement from the American Heart Association. Circulation, 149(3), e217-e231. 10.1161/cir.0000000000001189 PMC11209834 38059362

[bibr37-30495334251369446] Park H.-Y. Kim S. Kim Y. Park S. Nam S.-S. (2019). Effects of exercise training at lactate threshold and detraining for 12 weeks on body composition, aerobic performance, and stress related variables in obese women. Journal of Exercise Nutrition and Biochemistry, 23(3), 22–28. 10.20463/jenb.2019.0019 PMC6823647 31743978

[bibr38-30495334251369446] Porta A. Guzzetti S. Furlan R. Gnecchi-Ruscone T. Montano N. Malliani A. (2007). Complexity and nonlinearity in short-term heart period variability: Comparison of methods based on local nonlinear prediction. IEEE Transactions on Biomedical Engineering, 54(1), 94–106. 10.1109/TBME.2006.883789 17260860

[bibr39-30495334251369446] Rodrigues G. D. Dal Lago P. da Silva Soares P. P. (2021). Time-dependent effects of inspiratory muscle training and detraining on cardiac autonomic control in older women. Experimental Gerontology, 150, 111357. 10.1016/j.exger.2021.111357 33864832

[bibr40-30495334251369446] Roth G. A. Mensah G. A. Johnson C. O. Addolorato G. Ammirati E. Baddour L. M. Barengo N. C. Beaton A. Z. Benjamin E. J. Benziger C. P. Bonny A. Brauer M. Brodmann M. Cahill T. J. Carapetis J. Catapano A. L. Chugh S. S. Cooper L. T. Coresh J. . . . Fuster V. (2020). Global burden of cardiovascular diseases and risk factors, 1990-2019: Update from the GBD 2019 study. Journal of the American College of Cardiology, 76(25), 2982–3021. 10.1016/j.jacc.2020.11.010 33309175 PMC7755038

[bibr41-30495334251369446] Selig S. E. Carey M. F. Menzies D. G. Patterson J. Geerling R. H. Williams A. D. Bamroongsuk V. Toia D. Krum H. Hare D. L. (2004). Moderate-intensity resistance exercise training in patients with chronic heart failure improves strength, endurance, heart rate variability, and forearm blood flow. Journal of Cardiac Failure, 10(1), 21–30. 10.1016/s1071-9164(03)00583-9 14966771

[bibr42-30495334251369446] Shaffer F. Ginsberg J. P. (2017). An overview of heart rate variability metrics and norms. Frontiers in Public Health, 5, 258. 10.3389/fpubh.2017.00258 29034226 PMC5624990

[bibr43-30495334251369446] Shaffer F. McCraty R. Zerr C. L. (2014). A healthy heart is not a metronome: An integrative review of the heart’s anatomy and heart rate variability. Frontiers in Psychology, 5, 1040. 10.3389/fpsyg.2014.01040 PMC4179748 25324790

[bibr44-30495334251369446] Soares-Miranda L. Sattelmair J. Chaves P. Duncan G. E. Siscovick D. S. Stein P. K. Mozaffarian D. (2014). Physical activity and heart rate variability in older adults: The cardiovascular health study. Circulation, 129(21), 2100–2110. 10.1161/CIRCULATIONAHA.113.005361 24799513 PMC4038662

[bibr45-30495334251369446] Speer K. E. Semple S. Naumovski N. McKune A. J. (2020). Measuring heart rate variability using commercially available devices in healthy children: A validity and Reliability Study. European journal of investigation in health, psychology and education, 10(1), 390–404. 10.3390/ejihpe10010029 34542492 PMC8314243

[bibr46-30495334251369446] Stergiou N. (Ed.). (2020). Biomechanics and gait analysis (1st ed.). Elsevier.

[bibr47-30495334251369446] Task Force of the European Society of Cardiology and the North American Society of Pacing and Electrophysiology. (1996). Heart rate variability. Standards of measurement, physiological interpretation, and clinical use. Circulation, 93(5), 1043–1065. 10.1161/01.CIR.93.5.1043 8598068

[bibr48-30495334251369446] Tatro D. L. Dudley G. A. Convertino V. A. (1992). Carotid-cardiac baroreflex response and LBNP tolerance following resistance training. Medicine and Science in Sports and Exercise, 24(7), 789–796. 1501564

[bibr49-30495334251369446] Uthiravelu V. Bhavanani A. B. Hanifah M. Ramesh, & Krishnan J. (2023). Effect of 12 weeks yoga training and 12 weeks detraining on heart rate variability on subjects with hypertension. Journal of Advanced Zoology, 44(S-5), 1138–1143. 10.17762/jaz.v44is-5.1126

[bibr50-30495334251369446] Vallejo F. T. Chien L. T. Hébert-Losier K. Beaven M. (2020). Validity and reliability of the BeastTM sensor to measure movement velocity during the back squat exercise. The Journal of Sport and Exercise Science, 4(2), 100–105. 10.36905/jses.2020.02.05

