# Supplementary material for: Long-Term Impact of Physical Activity Levels After High-Speed Resistance Training on Cardiac Autonomic Control in Independent Older Adults
Source: Sage Open Aging. 2025 Aug 31;11:30495334251369446. doi: 10.1177/30495334251369446 (PMC12399819; doi:10.1177/30495334251369446)
Supplement: sj-docx-1-ggm-10.1177_30495334251369446 – Supplemental material for Long-Term Impact of Physical Activity Levels After High-Speed Resistance Training on Cardiac Autonomic Control in Independent Older Adults [file sj-docx-1-ggm-10.1177_30495334251369446.docx]

# **Table A:** Physical activity levels after the cessation of the 16-week high resistance training program.

| **Physical Activity Levels** | **Intervention** | | **Follow-up** | |
| --- | --- | --- | --- | --- |
|  | **M0 – Pre**  **N (%)** | **M1 – Post**  **N (%)** | **M2 – 6-Month**  **N (%)** | **M3 – 12-Month**  **N (%)** |
| Light | 11 (30.6) | 0 (0) | 10 (27.8) | 20 (55.6) |
| Moderate | 25 (69.4) | 4 (11.1) | 22 (61.1) | 11 (30.6) |
| Vigorous | 0 (0) | 32 (88.9) | 4 (11.1) | 5 (13.9) |

**Table B:** Distribution of self-reported physical activities at 12-month follow-up: light *vs.* moderate-to-vigorous intensity.

| **Groups** | **Activities self-reported** |
| --- | --- |
| LAG | 1 Participant – Occasionally walks at low intensity and yoga therapy  2 Participants – Once-a-week outdoor activity  4 Participants – Biweekly hydro gymnastic  6 Participants – Occasionally walks at low intensity  7 Participants – Did not perform any physical activity |
| MVAG | 1 Participant – 2–3 sessions per week of moderate-intensity continuous training, high-intensity interval training, and 2–3 walks longer than 40 min  1 Participant – Resistance training at least 2 times per week  2 Participants – Walk every day and biweekly hydro gymnastics  5 Participants – Walks longer 40 min every day  7 Participants – 2–3 sessions per week of moderate-intensity continuous training and high-intensity interval training |
| Abbreviatures: LAG, light activity group; MVAG, moderate-to-vigorous activity group. | |

**Table C:** General characteristics of the sample (Mean ± SD).

| **Measures** | **Groups** | **Intervention** | | **Follow-up** | | ***Interaction effect Within groups*** | ***Interaction effect Between groups*** |
| --- | --- | --- | --- | --- | --- | --- | --- |
|  |  | **M0**  **Pre** | **M1**  **Post** | **M2**  **6-Months** | **M3**  **12-Months** |  |  |
| Age (years) | LAG **^a,b,c^** | 68.55 ± 3.52 | 69.15 ± 3.57 | 69.45 ± 3.52 | 70.00 ± 3.66 | F=2.756¥  *p*=0.068  η²_p_=0.075* | F=0.013  *p*=0.908  η²_p_=0.001 |
|  | MVAG **^b,c,d,e^** | 67.31 ± 2.06 | 67.50 ± 2.09 | 68.19 ± 2.01 | 68.50 ± 2.09 |  |  |
| Weight (kg) | LAG | 67.89 ± 10.48 | 67.93 ± 11.37 | 66.64 ± 11.51 | 66.59 ± 11.01 | F=2.522¥  *p*=0.086  η²_p_=0.069* | F=0.110  *p*=0.742  η²_p_=0.003 |
|  | MVAG **^b,c,e^** | 68.68 ± 11.39 | 67.52 ± 11.90 | 66.32 ± 12.00 | 64.79 ± 11.89 |  |  |
| BMI (kg/m^2^) | LAG | 27.32 ± 4.32 | 27.31 ± 4.51 | 26.75 ± 4.47 | 26.68 ± 4.24 | F=1.650  *p*=0.182  η²_p_=0.046# | F=0.217  *p*=0.644  η²_p_=0.006 |
|  | MVAG **^b,c,e^** | 28.26 ± 3.57 | 27.76 ± 3.78 | 27.26 ± 3.88 | 26.58 ± 3.68 |  |  |
| Significant differences between periods are highlighted in bold (*p*≤ 0.05).  Abbreviations: LAG, light activity group; MVAG, moderate-to-vigorous activity group; Kg, kilograms; BMI, body mass index; m, meters.  ¥, Greenhouse-Geisser correction.  Significant differences:  a, pre-intervention *vs.* post-intervention;  b, pre-intervention *vs.* 6-month follow-up;  c, pre-intervention *vs.* 12-month follow-up;  d, post-intervention *vs.* 6-month follow-up;  e, post-intervention *vs.* 12-month follow-up;  f, 6-month follow-up *vs.* 12-month follow-up.  η_p_^2^ values thresholds:  #, small effect: 0.010 to 0.059;  *, medium effect: 0.060 to 0.140;  §, large effect large: > 0.140. | | | | | | | |


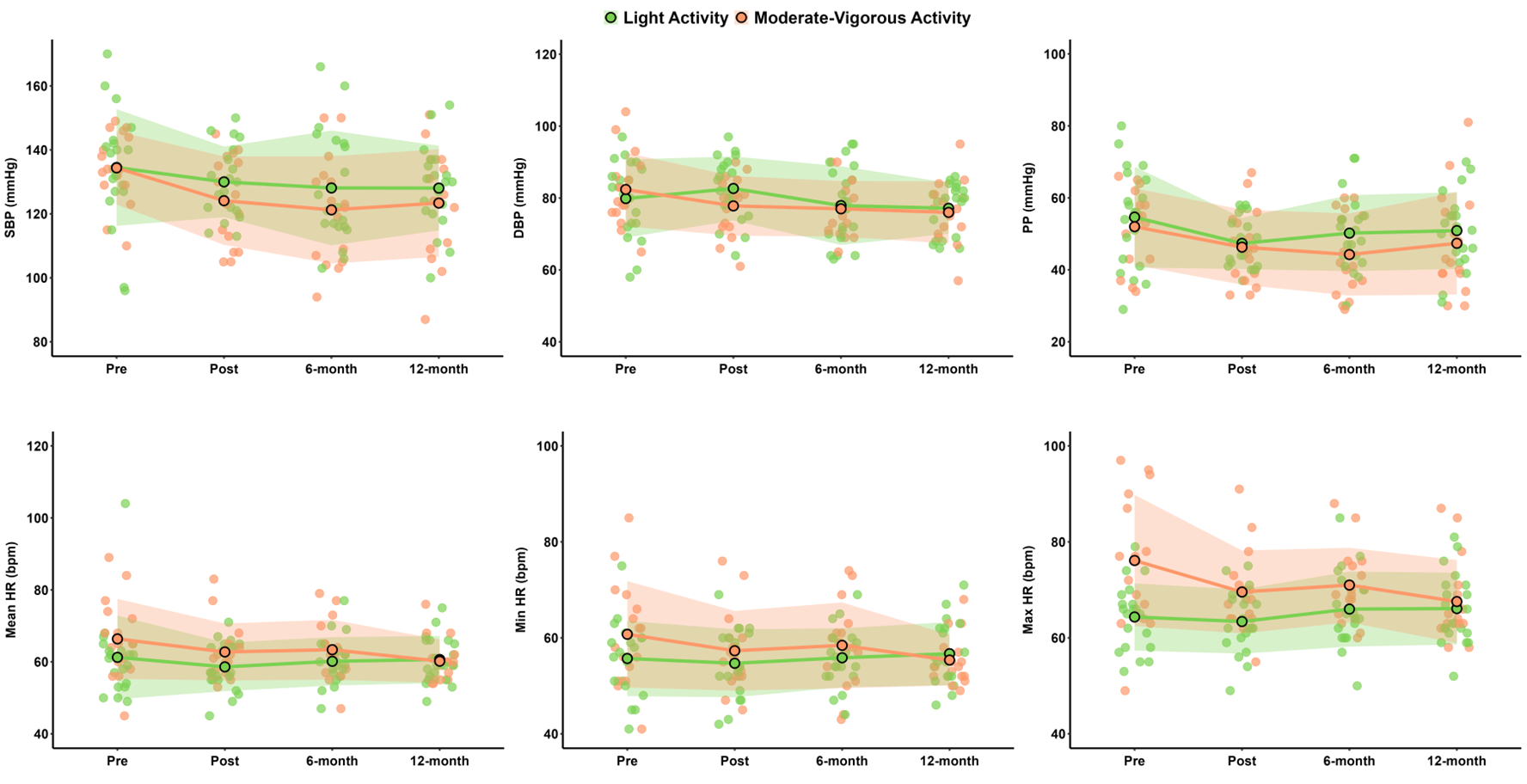


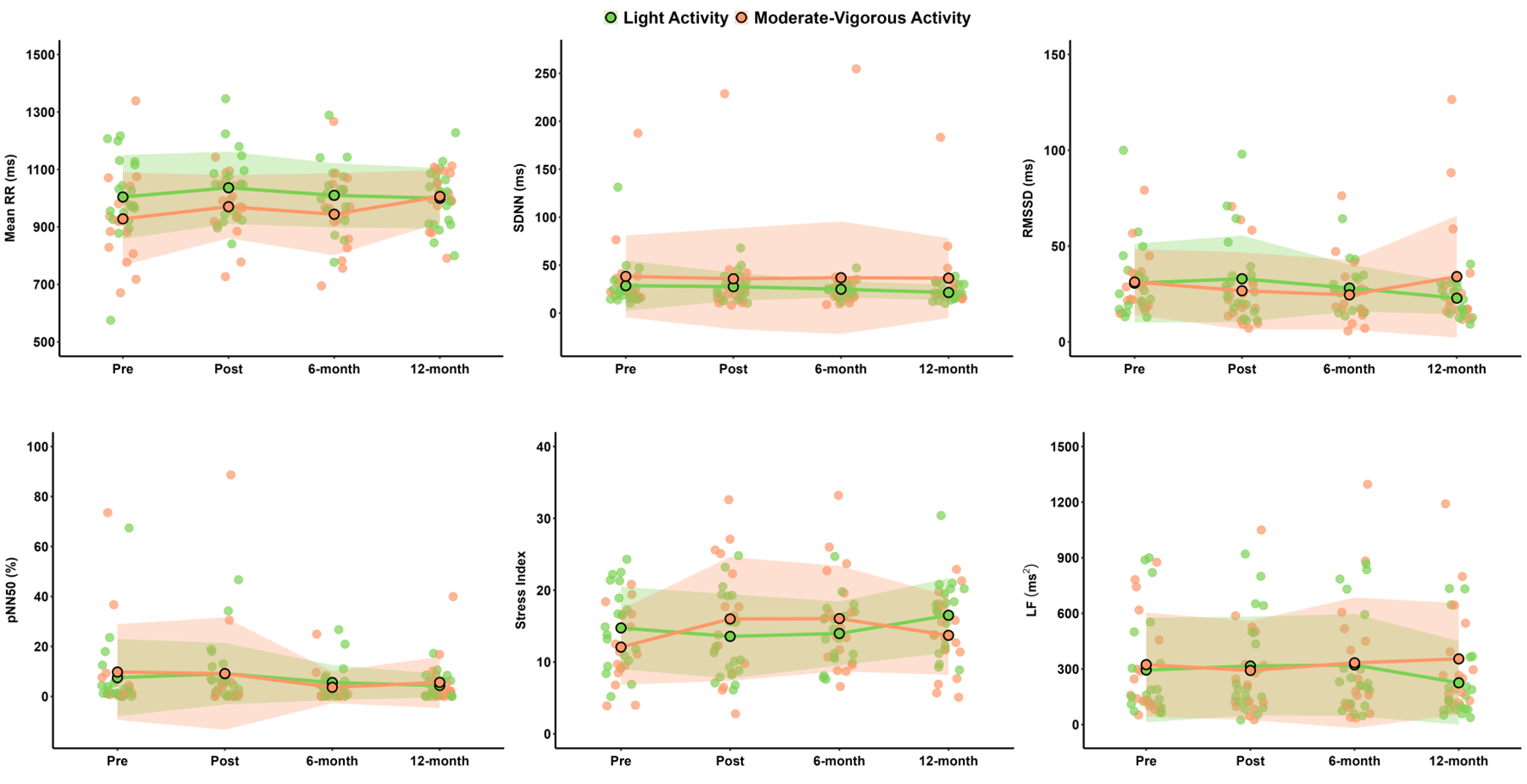


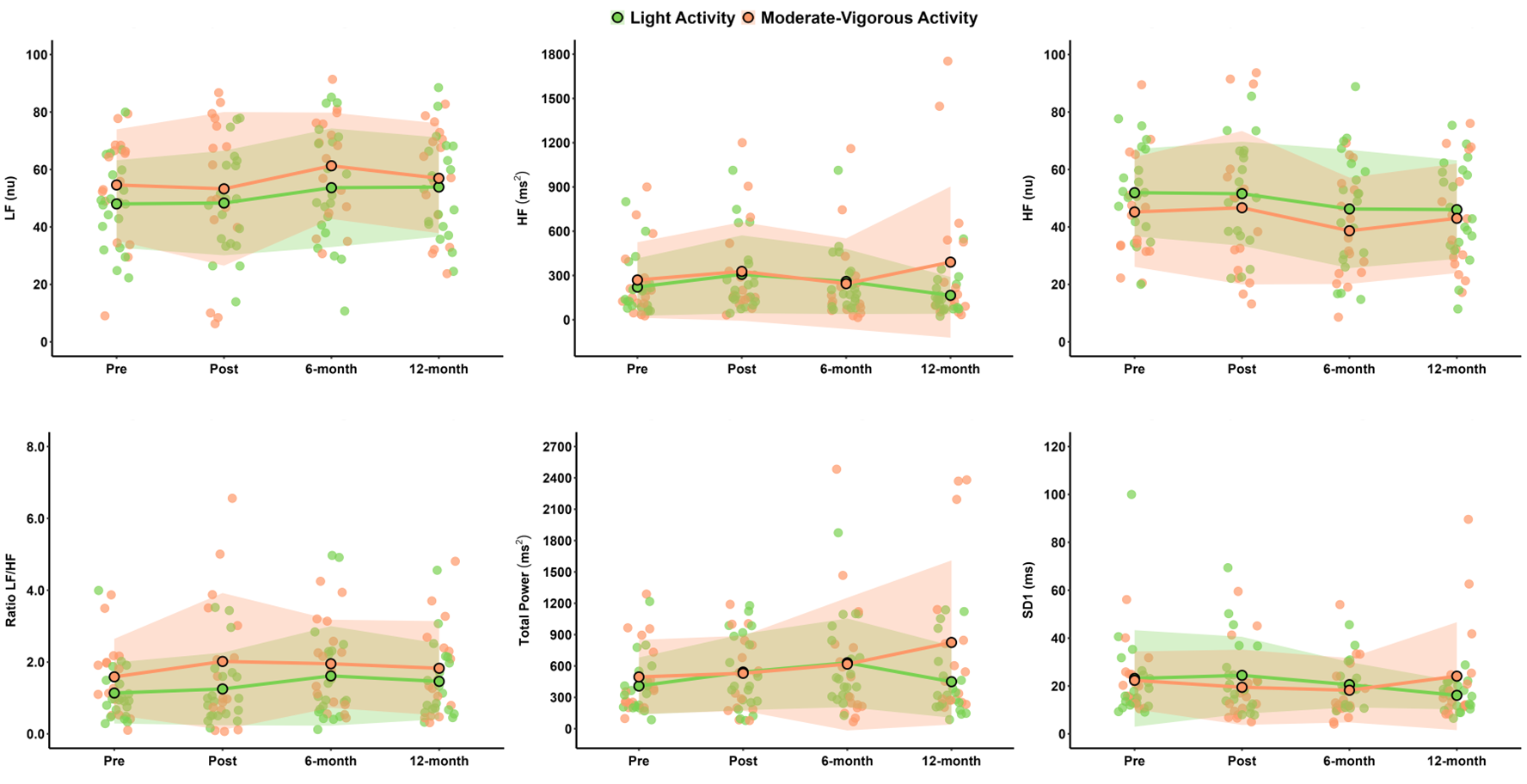


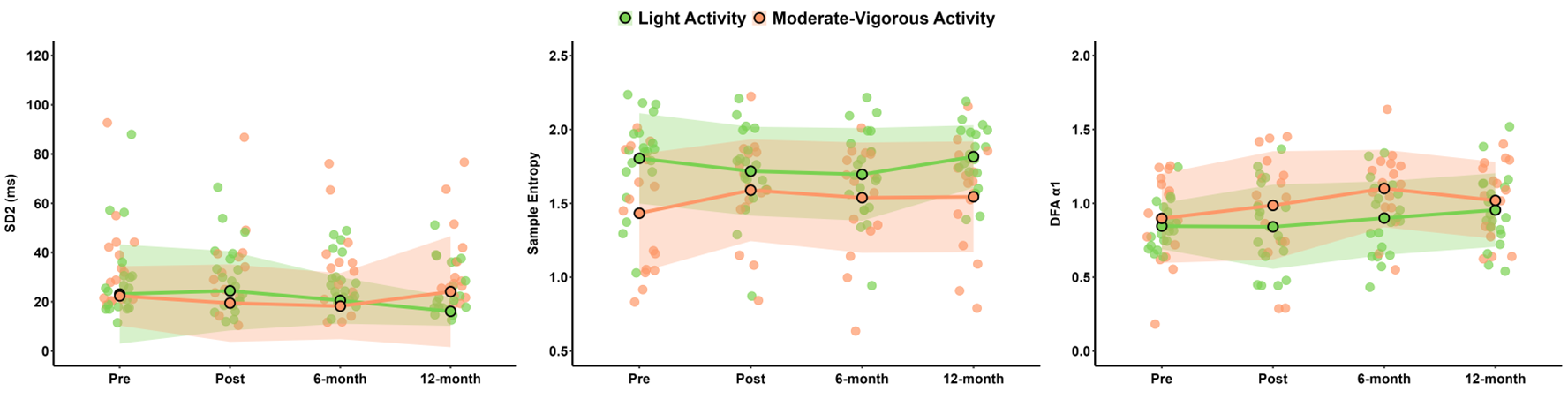


Figure S1: Heart rate variability indices for light activity and moderate-to-vigorous activity groups. Solid lines and filled dots represent mean values, while shaded areas indicate the standard deviation. Abbreviations: ms, milliseconds; SDNN, standard deviation of RR; RMSSD; root mean square of successive RR interval differences; pNN50, percentage of successive RR intervals differing by >50 ms; LF, low frequency; HF, High frequency; nu, normalized units; DFA, detrended fluctuation analysis.
